# Implementation of a hospital-wide EHR-integrated antibiotic challenge order set

**DOI:** 10.1017/ash.2025.10215

**Published:** 2025-10-27

**Authors:** Wesley Hoffmann, Shivani Patel, Shemual Tsai, Natalie Finch, Muhammad Yasser Alsafadi

**Affiliations:** 1 Department of Pharmacy, Houston Methodist Hospital, Houston, TX, USA; 2 Division of Infectious Diseases, Houston Methodist Hospital, Houston, TX, USA

## Abstract

We implemented an electronic health record-integrated order set to standardize direct antibiotic challenges for patients with reported antibiotic allergies across nine healthcare facilities. In the first year, 104 patients were challenged (79% oral; 39% two-step). Embedded antibiotic selection, dosage, monitoring parameters, reaction guidance, and rescue medications facilitated uptake by hospitalists and subspecialists.

## Introduction

Antibiotic allergy labels affect approximately 10% of hospitalized patients, yet over 90% of these labels are inaccurate when formally evaluated.^1 –3^ Inaccurate antibiotic allergy documentation leads to the unnecessary use of second-line or broader-spectrum antibiotics, higher healthcare costs, and worse clinical outcomes.^3–5^ Direct oral or intravenous antibiotic challenges in patients with low-risk allergy histories represent a safe and evidence-based strategy to clarify or remove inaccurate labels.^6–8^ However, real-world uptake has been limited due to operational and cultural barriers, including provider hesitation, inconsistent workflows, and time-consuming coordination with nursing and pharmacy staff.^2,3,5,9^

As part of a multifaceted quality improvement initiative to enhance antibiotic allergy assessment and management, we developed and implemented an electronic health record (EHR)-integrated antibiotic challenge order set across a multi-hospital health system. The purpose of this order set is to make it easier, safer, and more consistent for clinicians to perform direct oral or IV antibiotic challenges in patients labeled as antibiotic-allergic, especially when they are determined to be low-risk based on their allergy history.

Here, we describe its development, key design features, and early utilization experience.

## Methods

The quality improvement initiative was conducted across a nine-entity integrated healthcare system comprising an academic tertiary medical center, seven community hospitals, and one long-term acute care facility, all of which utilized a shared Epic EHR platform. The antibiotic challenge order set was developed collaboratively by infectious diseases physicians, pharmacists, informatics specialists, and an allergy physician. Iterative feedback from these developers and the antimicrobial stewardship team was incorporated prior to formal release.

**Figure 1.**
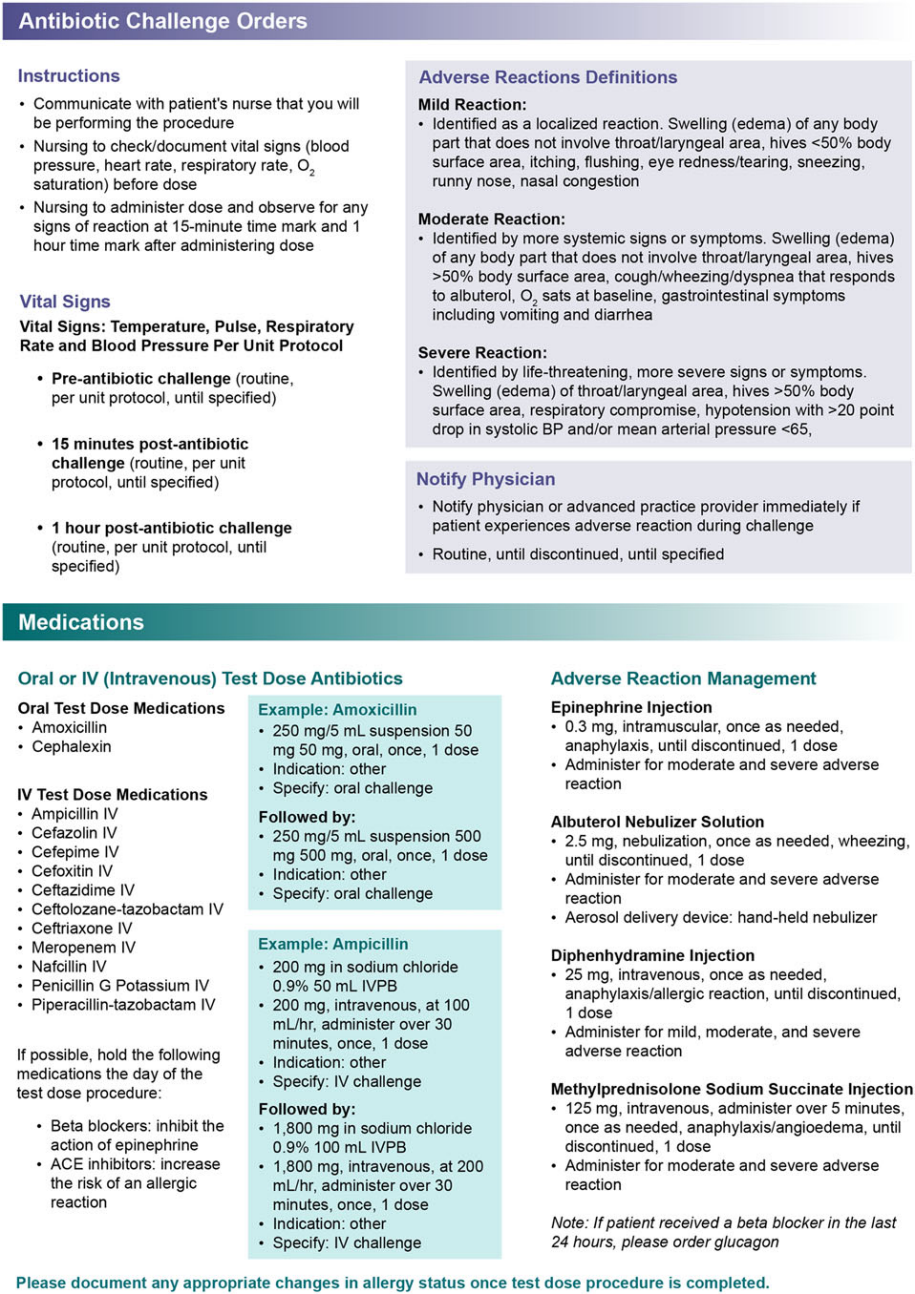
Structure of the EHR-integrated antibiotic challenge order set, including antibiotic agent selection, standardized single/two-step dosing options, nursing monitoring instructions, reaction definitions/next steps, as needed rescue medications, and allergy list update documentation prompt.

Figure 1 illustrates the structure and components of the EHR-integrated antibiotic challenge order set. The order set includes (1) selection of oral or IV antibiotic agents, (2) standardized test doses, (3) embedded nursing instructions and monitoring parameters, (4) definitions for mild, moderate, and severe reactions, and (5) preselected rescue medications. It is available to all credentialed prescribers and was promoted through committee newsletters, targeted provider e-mails, and real-time reinforcement by antimicrobial stewardship team members. Allergy risk assessments, patient selection, and order set use were at the discretion of the physician. Challenges may take place on any inpatient unit with bedside nursing monitoring per embedded instructions (vital signs prior to dosing and after each step; observation period specified in the order set). Rescue medications are preselected but not preadministered. Two-step challenges are administered 1 hour apart if the first dose was tolerated. The order set includes a prompt to update the EHR allergy list according to the outcome of the challenge; documentation is completed by the ordering team using an allergy activity update; a template is included with prespecified fields without direct stewardship oversight.

We conducted a retrospective descriptive analysis of the utilization of the order set for the initial 104 consecutive unique patients between June 28, 2024, and July 13, 2025. This project met criteria for quality improvement and did not require IRB oversight, consistent with institutional policy.

## Results

During the study period, the order set was utilized for 104 unique patients. A summary of patient and antibiotic challenge characteristics is provided in Table 1. Sixty patients were challenged at the academic tertiary medical center, and 44 at community hospitals. Of all the usages, 85 (82%) were ordered by infectious disease specialists, while the remaining were ordered by hospitalists.

**Table 1.** Patient and challenge characteristics

Characteristic	n. of patients (%)
Total unique patients	104 (100%)
Two-step challenges	41 (39%)
Antibiotic challenged*	
• Amoxicillin (PO)	78
• Ampicillin (IV)	6
• Cephalexin (PO)	5
• Cefazolin (IV)	2
• Cefepime (IV)	2
• Ceftazidime (IV)	1
• Ceftriaxone (IV)	4
• Meropenem (IV)	3
• Nafcillin (IV)	1
• Penicillin G (IV)	1
• Piperacillin-Tazobactam (IV)	2
Hospital type	
• Tertiary academic medical center	60 (58%)
• Community hospitals	44 (42%)
• Long-term acute care	0 (0%)
Ordering provider specialty	
• Infectious diseases	85 (82%)
• Hospitalists	19 (18%)
Route of antibiotic challenge	
• Oral	82 (79%)
• Intravenous	22 (21%)

* One patient was challenged with two antibiotics on separate occasions. IV, intravenous; PO, by mouth.

The most frequently challenged agents were amoxicillin (*n* = 78), ampicillin (*n* = 6), cephalexin (*n* = 5), and ceftriaxone (*n* = 4). Challenges were administered orally in 82 patients and intravenously in 22 patients. Forty-one patients (39%) received two-step challenges. Data on partial completions were not captured in this early implementation review.

## Discussion

This early implementation experience highlights the feasibility and practical utility of a standardized EHR-integrated order set for direct antibiotic challenges across a diverse hospital system. Uptake by both subspecialist and generalist providers suggests that the order set may help expand participation in antibiotic allergy de-labeling initiatives. Key facilitators included embedded monitoring instructions, as needed rescue medications, and standardized workflows that reduced ambiguity and supported clinical decision-making.

We did not report on patient demographics or outcomes, as the focus of this analysis was on operational rollout, provider uptake, and usability. Systematic safety monitoring was not conducted, consistent with the prespecified scope of this quality improvement initiative.

## Future directions

We plan to integrate the order set into our PEN-FAST-based allergy assessment workflow. This will involve linking low-risk scores to best practice alerts, prompting clinicians to use the antibiotic challenge order set and update allergy lists accordingly. We also aim to monitor process, balancing, and outcome metrics over time, including order set adoption, allergy list updates, and the change in antibiotic utilization trends.
